# Microtubule end tethering of a processive kinesin-8 motor Kif18b is required for spindle positioning

**DOI:** 10.1083/jcb.201705209

**Published:** 2018-07-02

**Authors:** Toni McHugh, Agata A. Gluszek, Julie P.I. Welburn

**Affiliations:** Wellcome Trust Centre for Cell Biology, School of Biological Sciences, University of Edinburgh, Edinburgh, Scotland, UK

## Abstract

Mitotic spindle positioning specifies the plane of cell division during anaphase. Spindle orientation and positioning are therefore critical to ensure symmetric division in mitosis and asymmetric division during development. The control of astral microtubule length plays an essential role in positioning the spindle. In this study, using gene knockout, we show that the kinesin-8 Kif18b controls microtubule length to center the mitotic spindle at metaphase. Using in vitro reconstitution, we reveal that Kif18b is a highly processive plus end–directed motor that uses a C-terminal nonmotor microtubule-binding region to accumulate at growing microtubule plus ends. This region is regulated by phosphorylation to spatially control Kif18b accumulation at plus ends and is essential for Kif18b-dependent spindle positioning and regulation of microtubule length. Finally, we demonstrate that Kif18b shortens microtubules by increasing the catastrophe rate of dynamic microtubules. Overall, our work reveals that Kif18b uses its motile properties to reach microtubule ends, where it regulates astral microtubule length to ensure spindle centering.

## Abstract (precis)

Kinesin-8 Kif18b shortens astral microtubules in mitosis. Combining cell biology and biochemical reconstitution, McHugh et al. show that Kif18b walks and accumulates to microtubule plus ends in a phosphospecific manner to regulate astral microtubule dynamics and center the mitotic spindle.

## Introduction

Spindle positioning and orientation is essential to ensure accurate chromosome partitioning and symmetrical cell division. Proper spindle placement is also particularly important during development and in stem-cell homeostasis, when cells divide asymmetrically to specify cell differentiation and generate daughter cells of different cell sizes and fates (Siller and Doe, 2009). The length and density of astral microtubules influence the position of the spindle by altering the interactions between astral microtubules and cortical force generators (Samora et al., 2011; Kiyomitsu and Cheeseman, 2012; Garzon-Coral et al., 2016). At the interphase-to-mitosis transition, the microtubule cytoskeleton undergoes rapid remodeling. The increased dynamism of microtubules allows the depolymerization of long interphase microtubules and subsequent assembly of dynamic spindle and astral microtubules that build and position the bipolar spindle (Belmont et al., 1990; Rusan et al., 2001).

Kinesin-8 and kinesin-13 motors regulate microtubule dynamics and length across eukaryotes. However, the microtubule depolymerization mechanism of kinesin-8 motors appears to differ across species. In budding yeast, Kip3 walks along microtubules and depolymerizes them (Gupta et al., 2006; Varga et al., 2009; Su et al., 2011), whereas *Drosophila melanogaster* Klp67A localizes to kinetochores, where it regulates spindle length (Savoian and Glover, 2010). Whether human kinesin-8 Kif18a motor is a depolymerizing enzyme, a processive motor that dampens microtubule plus-end dynamics, or both remains under debate (Mayr et al., 2007; Stumpff et al., 2008; Locke et al., 2017). A second human kinesin-8, Kif18b, is reported to exhibit diffusion on the microtubule lattice using its C terminus and weak directed motility, which does not explain how it could target to or destabilize microtubule plus ends (Shin et al., 2015). Kif18b has previously been implicated in the negative regulation of astral microtubule length and has a modest contribution to chromosome alignment (Stout et al., 2011; Tanenbaum et al., 2011; Walczak et al., 2016). Kif18b requires EB1 for microtubule end accumulation, but the EB-binding motifs in Kif18b are not sufficient for plus tip localization (Tanenbaum et al., 2011). Additionally, Kif18b may precede EB1 at microtubule ends (Shin et al., 2015), suggesting that other mechanisms enable Kif18b targeting to microtubule ends. Whether Kif18b cooperates with the kinesin-13 microtubule depolymerase mitotic centromere- associated kinesin (MCAK) or independently depolymerizes microtubule ends also remains under debate (Tanenbaum et al., 2011; Walczak et al., 2016).

In this study, we combine cell biology, biochemistry, and single-molecule reconstitution assays to define the molecular mechanisms that allow Kif18b to differentially target and accumulate at microtubule ends, where it plays an important role in regulating microtubule length and spindle positioning. We demonstrate that Kif18b tracks the growing ends of microtubules autonomously in vitro and reduces microtubule length by promoting microtubule catastrophe. We propose that Kif18b uses its motile properties to reach and accumulate at microtubule ends in a phosphospecific manner to selectively destabilize astral microtubules.

## Results

### Kif18b and MCAK are major mitotic motors negatively regulating microtubule length

Microtubule length regulation plays an important role in spindle assembly, geometry, and positioning. Previous work has analyzed the consequences of depleting kinesins that regulate microtubule length in human cells, but with differing results, possibly because of variable efficiencies of protein depletion or off-target effects (Manning et al., 2007; Mayr et al., 2007; Bakhoum et al., 2009; Tanenbaum et al., 2009; Welburn and Cheeseman, 2012). To identify kinesins that regulate microtubule length, we measured microtubule length in cells depleted for the kinesin-13 members Kif2a, Kif2b, and Kif2c/MCAK and the kinesin-8 members Kif18a and Kif18b using siRNA after Eg5 inhibitor treatment (Fig. S1, A and B). We found that both MCAK and Kif18b regulate microtubule length in mitotic cells, in agreement with previous studies (Stout et al., 2011; Tanenbaum et al., 2011; Walczak et al., 2016). However, Kif2a, Kif2b, and Kif18a depletion did not alter microtubule length, in agreement with previous studies (Tanenbaum et al., 2009; Welburn and Cheeseman, 2012). In addition, codepletion of Kif18b and MCAK did not have an additive effect on microtubule length, suggesting they may work together to regulate astral microtubules as previously suggested (Fig. S1, A and B; Tanenbaum et al., 2011).

**Figure 1.**
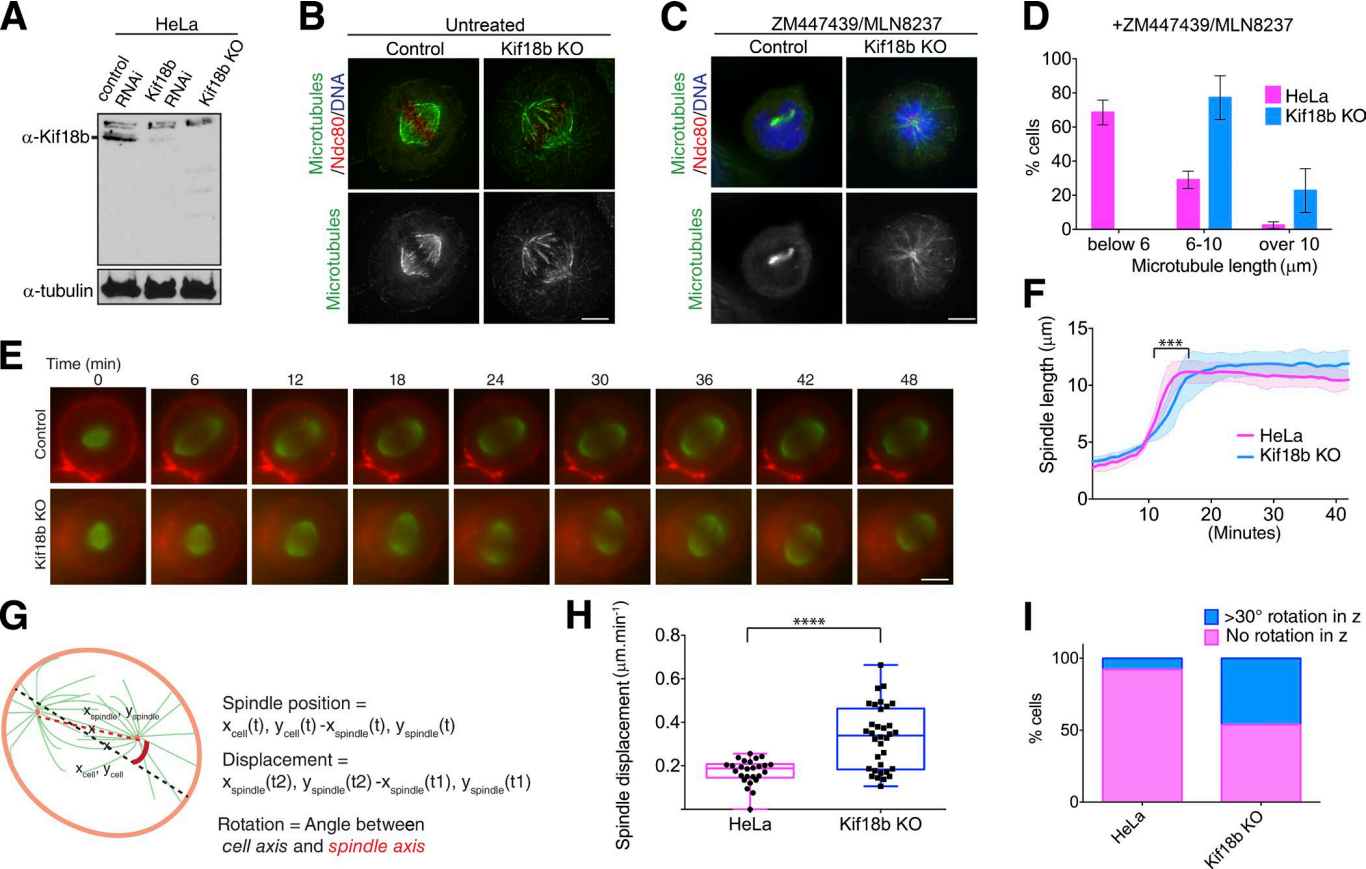
**Kif18b controls astral microtubule length and spindle positioning. (A)** Western blot showing depletion and gene KO of Kif18b in HeLa cells using siRNA and CRISPR-Cas9. **(B)** Representative immunofluorescence images of control and Kif18b-KO HeLa cells acquired using antibodies against Ndc80 and β-tubulin. **(C)** Representative immunofluorescence images of control and Kif18b-KO HeLa cells treated with Aurora kinase A and B inhibitors MLN8237 and ZM447439 acquired using antibodies against Ndc80 and β-tubulin. **(D)** Quantification of microtubule length was measured in >30 cells per experiment. Error bars represent SD. **(E)** Representative time-lapse imaging of cells incubated with SiR-tubulin and CellMask dye after an STLC washout and MG132 treatment. **(F)** Graph representing the mean spindle length and the corresponding SD for HeLa and Kif18b-KO HeLa cells (*n* = 32 and 36), defined in E during spindle elongation. Delay in spindle elongation of Kif18b-KO cells was statistically significant. **(G)** Schematic diagram showing how spindle position, displacement, and rotation are determined. **(H)** Box-and-whisker plot showing quantification of the spindle displacement from the center of the cell during metaphase. Each point represents the displacement of one spindle over at least 30 min (HeLa, *n* = 26 cells; Kif18b KO, *n* = 34 cells). Line in the middle box is plotted as the median, and edges of the box represent 25th and 75th percentiles. The whiskers represent the minimum and maximum displacement of cells. **(I)** Quantification of the number of cells undergoing >30**°** rotation in z out of the imaging plane during metaphase (HeLa, *n* = 86 cells; Kif18b KO, *n* = 122 cells). Bars, 5 µm. Asterisks indicate unpaired *t* test significance: ***, P < 0.001; ****, P < 0.0001.

To define the effect of Kif18b in regulating microtubule length, we generated a stable monoclonal HeLa cell line lacking Kif18b using CRISPR/Cas9-mediated gene targeting, indicating that Kif18b is not essential for viability of cultured HeLa cells (see Materials and methods). We found that Kif18b expression was eliminated from our monoclonal HeLa cell line targeted by Cas9 and offered an advantage in reproducibility over Kif18b siRNAi depletion, which needs to be performed for each experiment and may not result in 100% knockdown efficiency (Fig. 1 A). We then observed the mitotic microtubule organization of the cell line lacking Kif18b. As in cells depleted for Kif18b using RNAi, we observed a strong increase in the length of astral microtubules (Fig. 1 B; Stout et al., 2011; Tanenbaum et al., 2011). This was particularly apparent when Aurora kinases A and B were simultaneously inhibited. In the absence of Aurora kinase activity, microtubules in HeLa cells were almost completely depolymerized, whereas they remained >6 µm in the cell line lacking Kif18b (Fig. 1, C and D). This is consistent with the Aurora kinases negatively regulating Kif18b-mediated microtubule destabilization (Tanenbaum et al., 2011).

### Kif18b ensures correct spindle positioning

**Figure 2.**
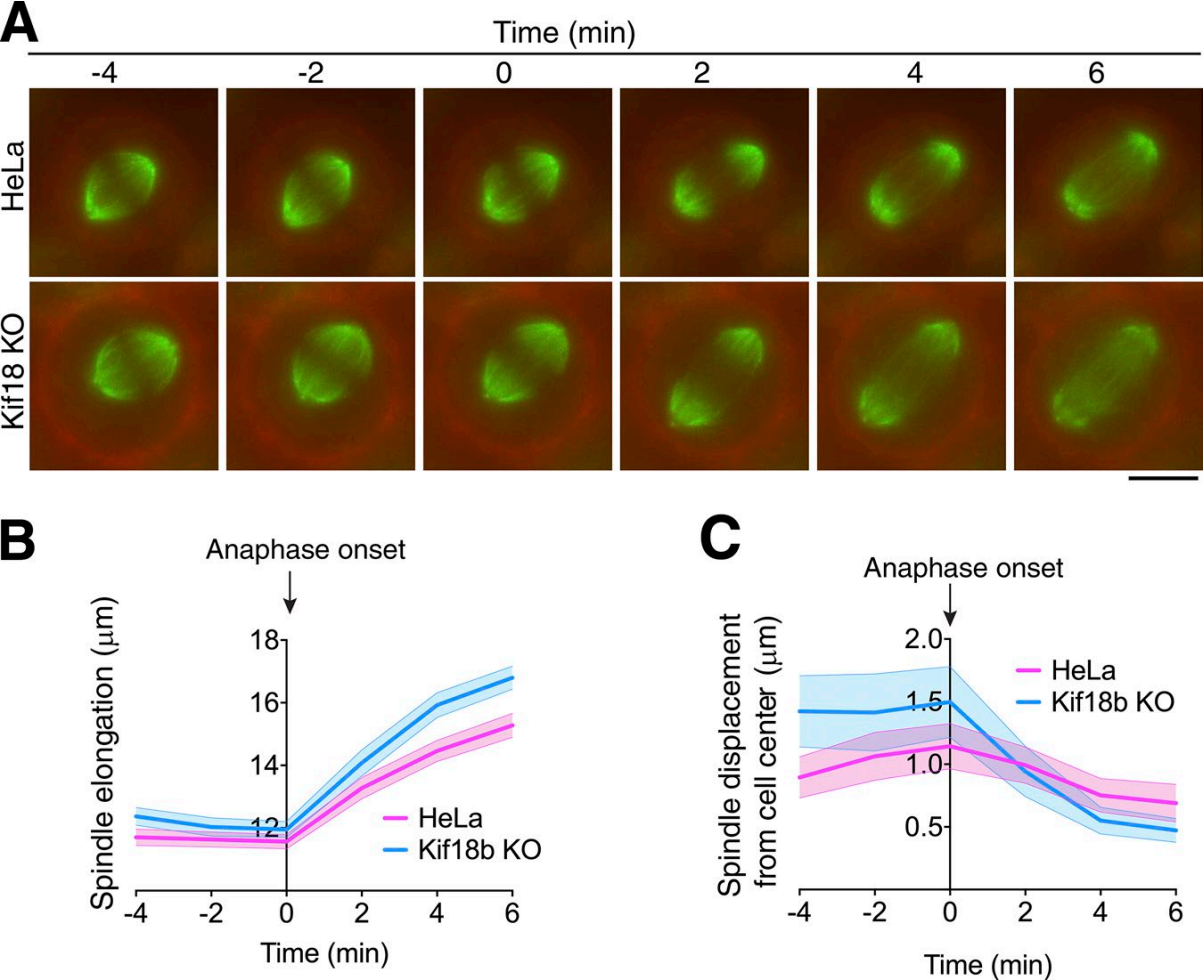
**Spindle centering is corrected during anaphase. (A)** Representative time-lapse images of cells going through mitosis incubated with SiR-tubulin (green) and CellMask dye (red). The 0 time point is defined as the last frame before anaphase spindle elongation takes place. Bar, 10 µm. **(B)** Graph representing the mean spindle length during the metaphase-to-anaphase transition for HeLa and Kif18b-KO HeLa cells (*n* = 64 and 44). **(C)** Graph showing the displacement of the center of the spindle with respect to the center of the cell along the spindle elongation axis. Shaded areas represent 95% confidence intervals.

To define the role of Kif18b-regulated astral microtubule length control on spindle assembly and positioning, we imaged mitotic cells using a previously described assay to examine spindle bipolarity establishment (Young et al., 2014). Overall, spindle elongation dynamics and length were similar in control cells and Kif18b knockout (KO) cells, although we observed a significant delay in reaching the maximum spindle length when Kif18b was absent (Fig. 1, E and F). The spindles of HeLa cells became bipolar and remained centered and parallel to the imaging plane, with a spindle displacement rate of 0.19 µm/min (±0.13 µm; Fig. 1, G and H). In contrast, we observed frequent spindle rotation outside of the imaging plane (>30**°**) in cells lacking Kif18b (Fig. 1 I). Kif18b KO spindles displayed strong positioning defects, with spindles moving around the cell center and proximally to the cell cortex (Video 1). Spindles had a random oscillatory-like trajectory, moving at a mean of 0.50 µm/min (±0.18 µm) within the cell (Fig. 1, G–I; and Video 1). Kif18b RNAi–depleted cells showed a similar spindle mispositioning phenotype, although the overall spindle length was similar to that of control cells (Fig. S1, C–E). These results indicate that increasing astral microtubule length causes spindle mispositioning and rocking, likely through additional interactions with force generators at the cell cortex. At anaphase entry, the spindles of Kif18b KO cells were less centered than those of control HeLa cells. During anaphase, the rate of spindle elongation and final spindle length for Kif18b KO cells were greater than those of control HeLa cells, possibly because of increased interactions of microtubules with cortical-force generators (Fig. 2, A and B). In both Kif18b KO cells and control HeLa cells, the displacement of the spindle with respect to the center of the cell decreased, resulting in the correction of spindle mispositioning (Fig. 2, A and C). Previous work has shown that an anaphase-specific dynein-dependent pathway centers the spindle as cells progress to anaphase (Kiyomitsu and Cheeseman, 2013). It is possible that the anaphase spindle positioning mechanism becomes essential when the length of microtubules increases and that the spindle is mispositioned during metaphase. Collectively, our data indicate that Kif18b plays a role in reducing the length of astral microtubules to facilitate the correct positioning of the metaphase spindle.

### Kif18b has a C-terminal microtubule-binding domain whose affinity is controlled by phosphorylation

**Figure 3.**
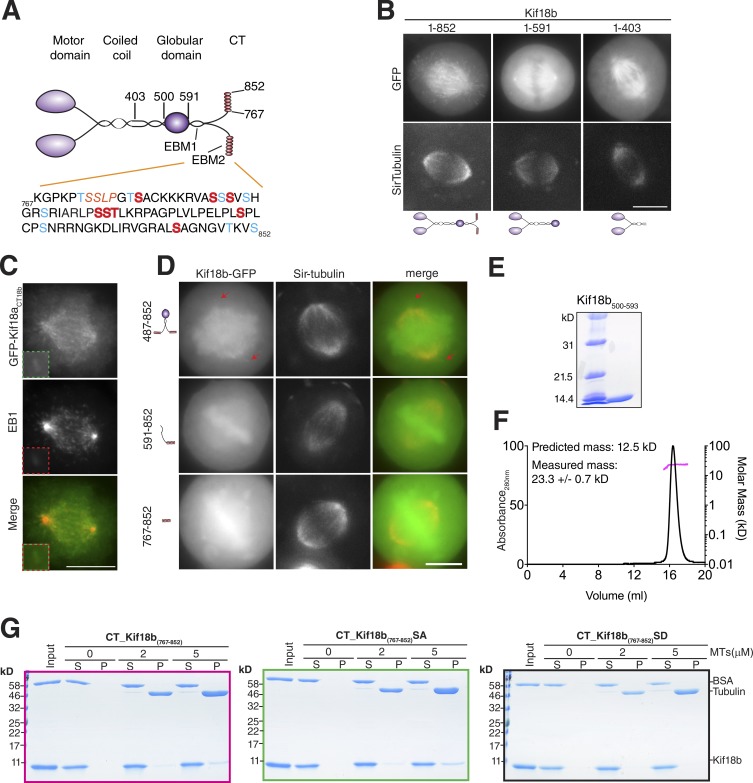
**The C terminus of Kif18b is a microtubule-binding domain. (A)** Top: Schematic diagram representing Kif18b structural domain organization. EB-binding motifs (EBMs) are indicated. Bottom: Amino acid sequence of the C terminus of Kif18b (CT_kif18b_). EB-binding motif is highlighted in orange. Red, amino acids that are phosphorylated in vivo and mutated to aspartates in this study; blue, phosphorylatable residues. **(B)** Representative live-cell images of HeLa cells transfected with full-length and truncated Kif18b motor, costained with Sir-tubulin. **(C)** Representative images by immunofluorescence of chimeric construct GFP-Kif18a_CT18b_ (motor domain of Kif18a fused to the C terminus of Kif18b) costained with an anti-EB1 antibody. **(D)** Representative live-cell images of truncation constructs of Kif18b C terminus. Red arrows indicate Kif18b_487–852_ localizing to microtubule (MT) plus ends. Bar, 10 µm. **(E and F)** Coomassie-stained gel showing recombinantly expressed and purified Kif18b_500–593_ (E) and its corresponding elution profile (F; black line, left y axis) from SEC-MALS analysis. Outcome of the MALS analysis for the peak is presented in pink (molecular weight, right y axis). **(G)** Coomassie-stained gels showing a cosedimentation assay of CT_Kif18b_, CT_Kif18b_SA, and CT_Kif18b_SD in the presence of 2 µM taxol-stabilized microtubules.

Kif18b localizes to microtubule ends during mitosis (Tanenbaum et al., 2011; Walczak et al., 2016). Although Kif18b requires EB1 to localize to microtubule ends, additional factors play a role in Kif18b plus-end targeting (Stout et al., 2011; Tanenbaum et al., 2011). To define the unique targeting properties of Kif18b, we generated multiple Kif18b constructs based on its molecular organization (Fig. 3 A). First, we observed that the GFP N-terminal tagging of Kif18b led to chromosome targeting of Kif18b as well as its physiological localization to astral microtubules (Fig. S1 F). This indicates that GFP at the N terminus of Kif18b interferes with its motor properties. We then generated minimal Kif18b motor constructs lacking the C terminus with a C-terminal GFP. Kif18b-GFP displayed robust localization to microtubule plus ends (Fig. 3 B). In the absence of the C terminus, the Kif18b motor targets only to spindle microtubules (Fig. 3 B). To test whether the C terminus of Kif18b was the main determinant in motor targeting, we swapped the C termini of Kif18a for those of Kif18b. Despite highly conserved motor domains, Kif18a and Kif18b target to distinct cellular localizations. Typically, Kif18a targets to K-fibers and kinetochores, whereas Kif18b targets to microtubule plus ends (Fig. 3 B; Mayr et al., 2007). We generated a chimeric kinesin consisting of the motor and neck-linker region of Kif18a fused to the C terminus of Kif18b. This Kif18a-Kif18b chimera targeted to microtubule ends, colocalizing with EB1 (Fig. 3 C), in contrast with the spindle localization of the motor domain of Kif18a alone (Stumpff et al., 2011). These experiments demonstrate that the C terminus of Kif18b determines the localization of the motor domain to microtubule ends and that the Kif18a motor domain can become a Kif18b-like motor when fused to the C terminus of Kif18b.

We next examined the role of the C terminus of Kif18b in Kif18b targeting. The C terminus is largely unstructured, except for domain 500–593, which is predicted to be globular. In cells, Kif18b_500–852_ targeted weakly to microtubule ends (Fig. 3 D). Surprisingly, Kif18b_591–852_ targeted to the spindle, rather than microtubule ends. This result suggests that this region contains a second weak microtubule-binding region, but also that Kif18b_500–593_ may promote C terminus dimerization to increase the affinity of Kif18b for EB proteins and microtubules (Fig. 3 D). Indeed, we found that Kif18b_500–593_ is a dimer in solution (Fig. 3, E and F). To test whether the C terminus of Kif18b could bind microtubules independently of the motor, we generated a series of truncated constructs and found that the far C-terminal tail (CT; CT_Kif18b_, amino acids 767–852), is sufficient to localize to spindle microtubules (Fig. 3 D). In addition, we found that recombinant CT_Kif18b_ binds to microtubules in vitro (Figs. 3 G and S2). The isoelectric point of the CT_Kif18b_ is 11.92, highly basic and likely to bind electrostatically to the negatively charged microtubule. This also explains why the Kif18b C terminus binds to DNA in vivo (Fig. 3 D).

The CT_Kif18b_ is strongly phosphorylated in human cells during mitosis (Fig. 3 A; Dephoure et al., 2008). To test whether phosphorylation regulates the microtubule binding properties of the CT_Kif18b_, we generated a phosphomimetic mutant by substituting eight residues within Kif18b_767–852_ that are phosphorylated in vivo to aspartates (Figs. 3 A and S2; Dephoure et al., 2008; Sharma et al., 2014). This phosphomimetic CT_Kif18b_SD did not bind to microtubules in vitro, but a nonphosphorylatable mutant CT_Kif18b_SA did, similarly to CT_Kif18b_ (Fig. 3 G). These data indicate that phosphorylation regulates the affinity of the CT_Kif18b_ for microtubules by changing the electrostatic charges of the CT_Kif18b_. The CT_Kif18b_ contains additional serines and threonines, which could be phosphorylated, although they have not been identified so far. Collectively, these results show that the C terminus of Kif18b provides a second microtubule-binding domain to Kif18b that is regulated by phosphorylation.

### The Kif18b C terminus is required for Kif18b accumulation at microtubule plus ends

**Figure 4.**
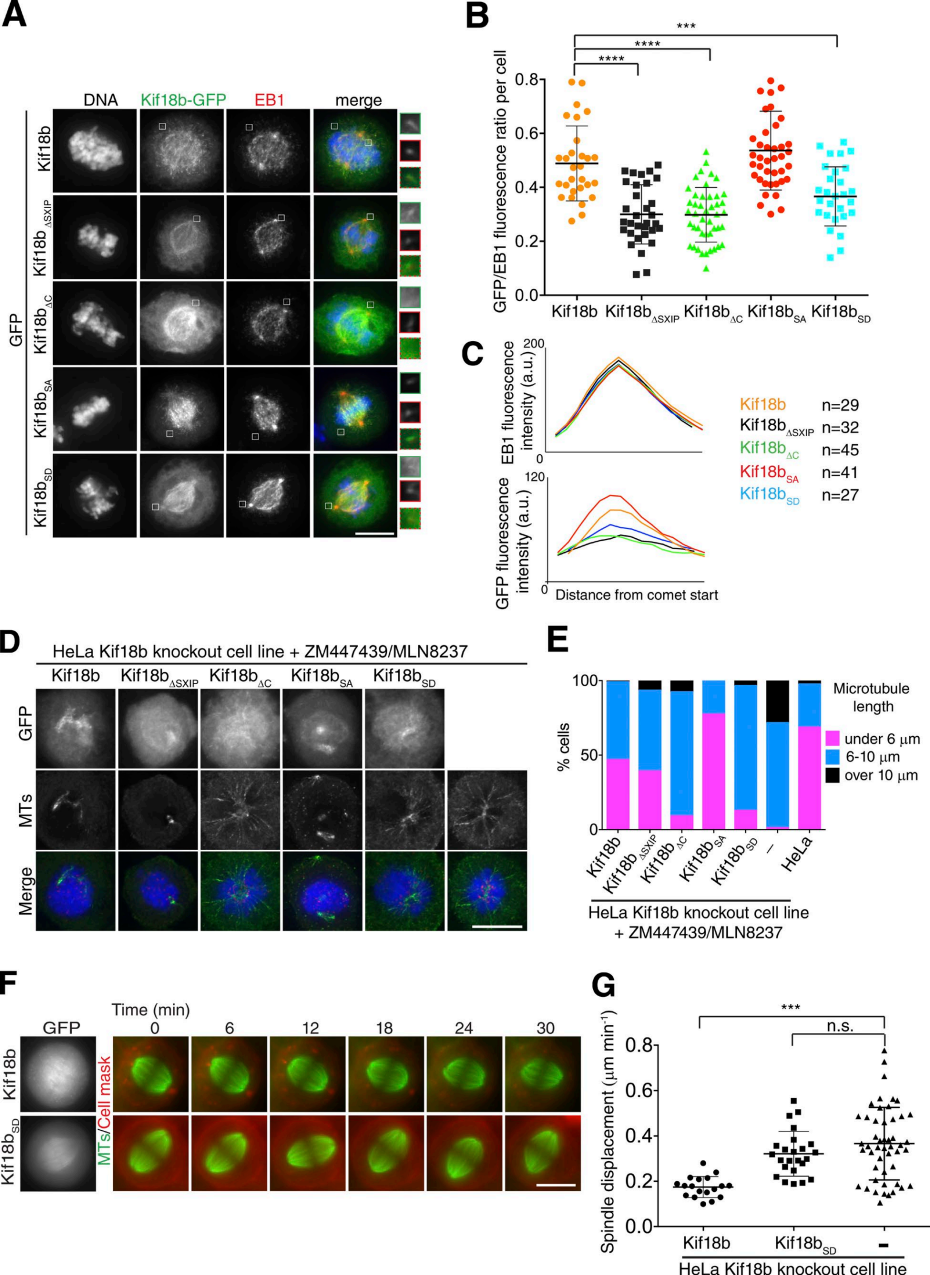
**The C terminus of Kif18b contributes to Kif18b targeting to microtubule plus ends and the control of microtubule length in vivo. (A)** Representative immunofluorescence images of Kif18b-KO HeLa cells transfected with Kif18b-GFP constructs and stained with an anti-EB1 antibody. **(B)** Quantification of GFP/EB1 signal intensity ratio at microtubule (MT) plus ends. Each data point represents one cell for which a mean of four comet intensities were measured and averaged (mean ± SD). Kif18b-GFP transfection was repeated twice; all others were repeated three times. **(C)** Mean linescan profile of measured EB1 comets and their corresponding linescan profiles for GFP-Kif18b constructs from cells measured in B. **(D)** Representative immunofluorescence images of Kif18b-KO HeLa cells transfected with Kif18b constructs, stained with Hoechst for DNA, and immunostained with anti-Ndc80 and antitubulin antibodies. Cells were treated with the Aurora kinase A and B inhibitors MLN8237 and ZM447439. **(E)** Quantification of microtubule length in cells treated with STLC and MLN8237/ZM447439 after transfection of Kif18b-GFP constructs (111 > *n* > 66). **(F)** Time-lapse imaging series of Kif18b-KO HeLa cells transfected with Kif18b-GFP and Kif18b_SD_-GFP, imaged with SiR-tubulin and CellMask dye. **(G)** Quantification of the displacement of the spindle from the center of the cell during metaphase (mean ± SD). Each data point represents spindle displacement over 30–40 min. Asterisks indicate ordinary one-way ANOVA significance value: ***, P < 0.001; ****, P < 0.0001. Bars, 10 µm.

Because Kif18b has a C-terminal nonmotor microtubule-binding domain, CT_Kif18b_, we hypothesized that it may regulate the microtubule end targeting and functional properties of full-length Kif18b in vivo. To distinguish the contribution of the microtubule-binding properties of the CT_Kif18b_ and the end-binding (EB) motifs to the plus end accumulation of Kif18b in vivo, we transfected Kif18b, Kif18bΔ_SXIP_ mutated for the last two residues in the putative EB-binding motif to NN (Aps-Asp), and Kif18bΔ_C_, a mutant lacking the CT_Kif18b_ into the Kif18b KO cells. Kif18b colocalized with EB1 at microtubule plus ends (Fig. 4, A–C). Kif18bΔ_SXIP_ had strongly reduced plus tip localization. However, we could still observe weak plus tip targeting (Fig. 4, A–C) as previously reported (Tanenbaum et al., 2011). Kif18bΔ_C_ was also strongly reduced at microtubule plus ends (Fig. 4, A–C), despite the presence of an upstream SxIP motif (Fig. 3 A). Importantly, Kif18b_SXIPmut2_, with mutations in the C-terminal EB-binding motif (_773_SXIP_776_), still robustly localized to microtubule ends (Figs. 3 A and S3, A and B), indicating that the CT_Kif18b_ and the SxIP-binding motifs play nonredundant targeting functions.

Because mitotic phosphorylation decreases the affinity of the CT_Kif18b_ for microtubules, we hypothesized that phosphorylated Kif18b would accumulate less at astral microtubule plus ends, whereas a nonphosphorylatable Kif18b mutant would be enriched. To test this, we generated nonphosphorylatable (Kif18_SA_-GFP) and phosphomimetic (Kif18_SD_-GFP) mutants for the eight previously identified phosphorylated sites (Dephoure et al., 2008). Kif18b_SA_ targeting to astral microtubule plus tips was comparable with that of Kif18b. We could also see some accumulation of Kif18b_SA_ to microtubule ends within the spindle (Fig. 4 A). In contrast, Kif18b_SD_ showed severely reduced astral plus tip localization, similar to Kif18bΔ_C_ and Kif18bΔ_SXIP_, indicating that mitotic phosphorylation of its CT_Kif18b_ controls its accumulation at astral microtubule ends (Fig. 4, A–C). Collectively, these results indicate that Kif18b uses both its C-terminal microtubule-binding properties and its EB-binding motifs to accumulate at microtubule plus ends. Phosphorylation of the CT_Kif18b_ reduces its plus-end accumulation, which could control the spatial distribution of Kif18b during mitosis.

### The C terminus of Kif18b is a requirement for microtubule length control and spindle positioning

Next, we sought to test whether the CT_Kif18b_ was necessary for its ability to shorten astral microtubules and center the spindle in Kif18b KO cells. First, we tested that Kif18b-GFP could restore astral microtubule length in Kif18b KO cell lines (Fig. S3, C and D). However, it is challenging to measure astral microtubule length. Therefore we used the robust assay previously performed in Fig. 1 (C and D) to test the depolymerase activity of our Kif18b mutants in the presence of Aurora kinase small molecule inhibitors. In our HeLa Kif18b KO cell line, microtubules remain long in the presence of Aurora kinase inhibitors (Fig. 1, C and D). Kif18b and Kif18b_SA_ transfection restored the control of microtubule length, with shortened and depolymerized microtubules as observed in HeLa cells (Fig. 4, D and E). In contrast, the ability of Kif18b_SD_ and Kif18bΔ_C_ to depolymerize microtubules was low, suggesting that CT_Kif18b_ plays a role in microtubule depolymerization. Kif18bΔ_SXIP_ displayed an intermediate ability to depolymerize microtubules, consistent with its weak localization to plus ends (Fig. 4 E). Finally, we tested whether the CT_Kif18b_ was important for the ability of the kinesin to control spindle positioning. Kif18b restored correct spindle centering to cells lacking Kif18b, in line with its ability to reduce astral microtubule length (Fig. 4, F and G; and Fig. S3, C and D). However, the ability of Kif18b_SD_ to center the spindle was strongly reduced, consistent with the fact that Kif18b_SD_ did not rescue proper microtubule length. These results indicate that the presence of the CT_Kif18b_ is important for microtubule length regulation and spindle positioning. Phosphorylation of the CT_Kif18b_ reduces Kif18b accumulation at microtubule ends to prevent microtubule depolymerization.

### Kif18b is a processive plus end–directed motor

**Figure 5.**
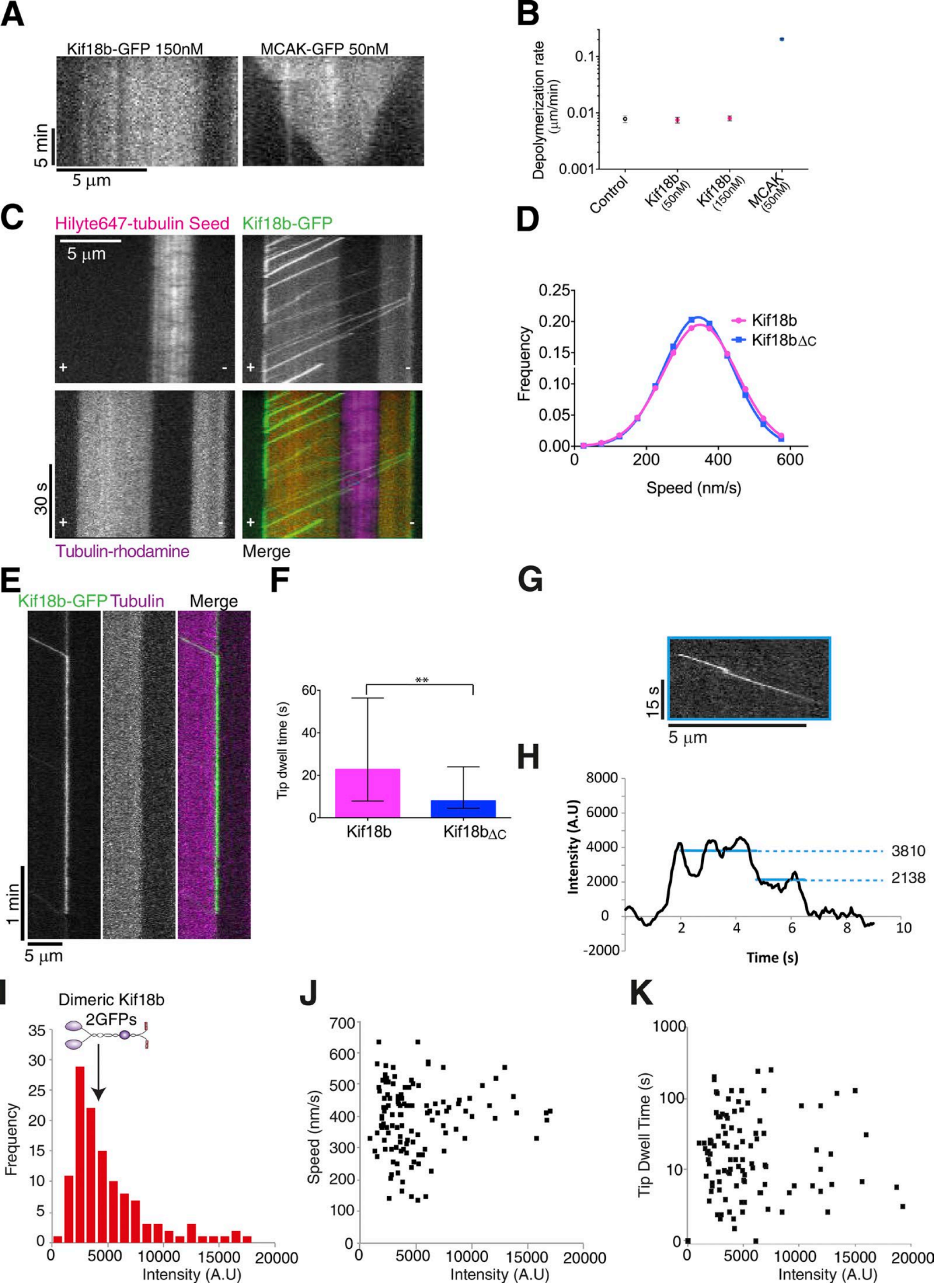
**Kif18b is a fast processive plus end–directed motor that uses its C terminus to accumulate at microtubule ends. (A)** Example kymographs of GMP-CPP–stabilized microtubules in the presence of 150 nM Kif18b and 50 nM MCAK. **(B)** Depolymerization rates for GMP-CPP–stabilized microtubules with no added motor (*n* = 47), 50 nM Kif18b (*n* = 74), 150 nM Kif18b (*n* = 50), and 50 nM MCAK (*n* = 113; mean ± SEM). **(C)** Kif18b is a plus end–directed motor with a run length of >7 µm. Polarity-marked GMP-CPP–stabilized microtubules (HiLyte and rhodamine labeled) with Kif18b motors. Motors walk processively toward the plus end of the microtubules, pausing at the microtubule tips before dissociation. **(D)** Velocities of Kif18b (*n* = 193) and Kif18bΔ_C_ (*n* = 142) as measured from kymographs. **(E)** Example kymograph showing Kif18b dwelling at a microtubule tip for >1 min. **(F)** Tip dwell time for Kif18b (*n* = 103) and Kif18bΔ_C_ (*n* = 73). Kif18bΔ_C_ dwells at microtubule tips for shorter times than full-length Kif18b (median; error bars represent interquartile range). **, P < 0.01. **(G)** Kymograph showing a processively moving Kif18b-GFP motor that photobleaches in two steps, indicating the presence of two GFP molecules. **(H)** Corresponding intensity profiles for motors in G. Horizontal lines show the mean intensity for the respective sections of the profile. **(I)** Frequency of intensity of Kif18b-GFP motors at the beginning of a processive run; the majority of motors form a peak at 3,500 AU. However, ∼35% of motors have intensities >5,000 AU. **(J)** Dependence of velocity on intensity for Kif18-GFP motors; no clear dependence on intensity can be seen. **(K)** Dependence of tip dwell time on intensity for Kif18-GFP motors; no clear dependence on intensity can be seen.

The precise activity of Kif18b and how it reaches microtubule ends to shorten them remain unclear. To determine whether Kif18b acts as a bona fide microtubule depolymerase at microtubule ends, we expressed and purified Kif18b-GFP from insect cells (Fig. S4 A). Using size-exclusion chromatography–multiangle light scattering (SEC-MALS), we determined that Kif18b-GFP assembles as a dimer in solution (Fig. S4 B). We immobilized GMPCPP-stabilized microtubules on coverslips that had surface-adsorbed antitubulin antibodies at low density. Microtubules alone remained stable over time. In the presence of 50 nM MCAK, microtubules rapidly depolymerized at a speed of 0.2 µm/min. In contrast, in the presence of Kif18b, the microtubules remained stable (Fig. 5, A and B). This suggests that Kif18b does not use the same mechanism as MCAK and Kip3 to depolymerize microtubules (Gardner et al., 2011; Su et al., 2011). To analyze the motile properties of Kif18b, we imaged Kif18b-GFP on stabilized microtubules using a single-molecule total internal reflection fluorescence (TIRF) microscopy. We observed that Kif18b moved processively toward the plus ends of microtubules for long distances, with a mean speed of 349 ± 7 nm/s (mean ± SEM; Fig. 5, C and D; and Video 2). Kif18b could travel over distances >7 µm, with the run length limited by the length of the microtubule (Fig. S4, C and D). As Kif18b-GFP reached the plus end of microtubules, it dwelled at the plus tip of microtubules for a significant amount of time (Fig. 5, E and F). Thus Kif18b accumulates at the end of stabilized microtubules in vitro independently of EB proteins. In our single-molecule imaging, we observed variable GFP intensities of moving Kif18b. To test the oligomeric composition of Kif18b, we used single-molecule photobleaching and found that most Kif18b molecules were dimeric (Fig. 5, G–I). We did see variability in GFP intensities, suggesting that some Kif18b-GFP molecules oligomerize on microtubules over time (Fig. 5 C). However, the intensity of Kif18b-GFP molecules did not correlate with the speed or tip dwell time of Kif18b (Fig. 5, J and K). These data indicate that Kif18b is a fast and processive plus end–directed motor that accumulates at the ends of microtubules in vitro*.*

Given that CT_Kif18b_ is important for its accumulation at microtubule ends in vivo*,* we hypothesized it may be important for this targeting property in vitro. To test the role of the CT_Kif18b_ on the motility and microtubule end targeting of full-length Kif18b, we purified Kif18bΔ_C_-GFP lacking the last 75 amino acids of the CT_Kif18b_. Kif18b and Kif18bΔ_C_ had a similar elution profile (Fig. S4 E). Overall, the velocity of Kif18bΔ_C_ was similar to that of Kif18b (Fig. 5, C and D), and the motor remained highly processive (Fig. S4, C and D). However, there was a remarkable reduction in its dwell time at the tips. On average, Kif18bΔ_C_ remained at the plus end of microtubules 8.5 s, in contrast with Kif18b (22.8 s; Fig. 5 F). In total, our data indicate that the CT_Kif18b_ plays a key role in the tethering of Kif18b, independently of EB1, once Kif18b reaches the microtubule end.

### The C terminus of Kif18b interferes with motor domain depolymerase activity

**Figure 6.**
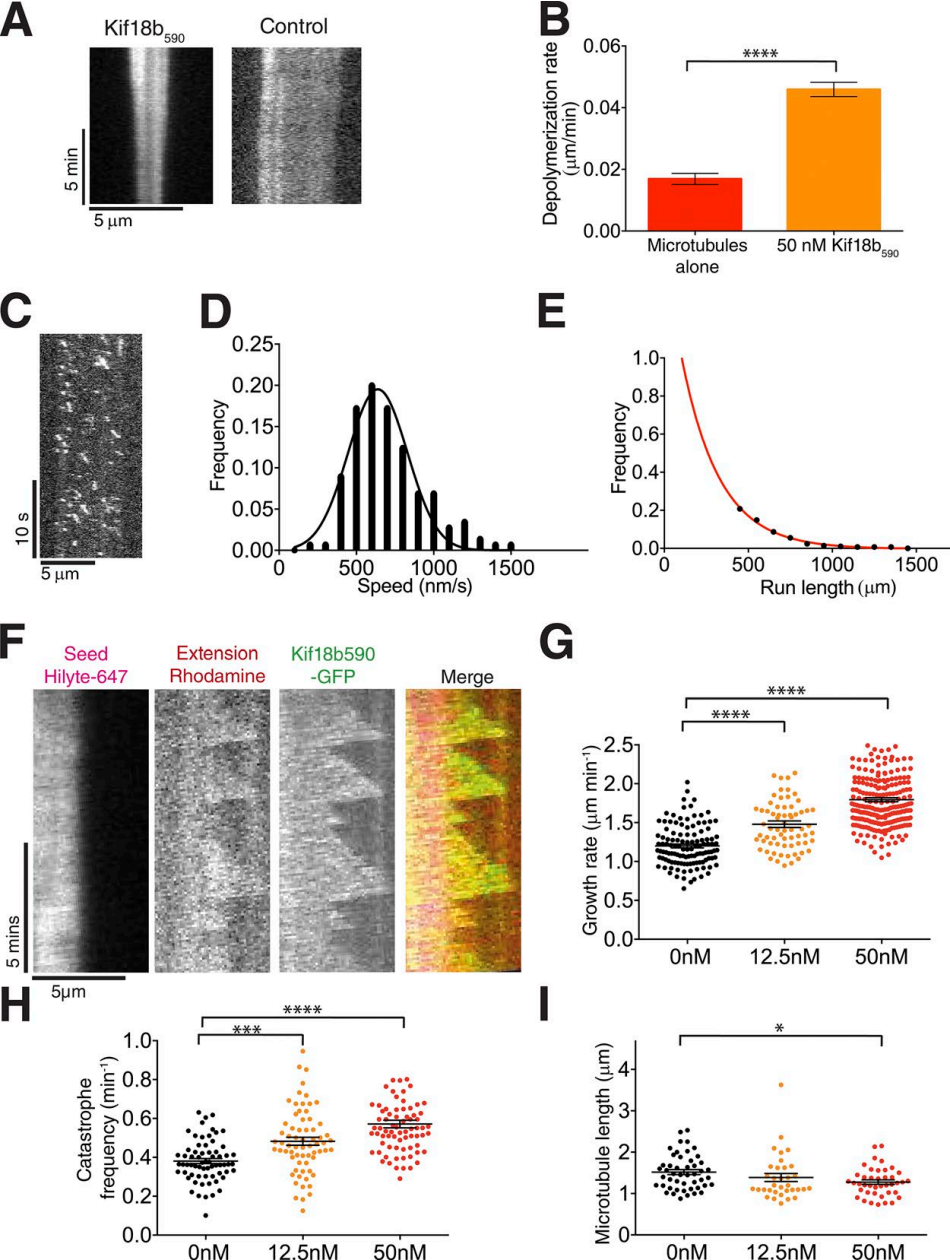
**Kif18b_590_ depolymerizes microtubules. (A)** Example kymographs of GMP-CPP–stabilized microtubules in the absence and presence of 50 nM Kif18b. **(B)** Depolymerization rates for GMP-CPP–stabilized microtubules with no added motor (*n* = 97) and 50 nM Kif18b (*n* = 125; mean ± SEM). Unpaired *t* test significance: ****, P < 0.0001. **(C)** Example kymograph of Kif18b_590_ in 0.1 mM ATP. **(D)** Histogram plot of Kif18b_1–590_ velocities determined from kymograph in A; *n* = 229. **(E)** Run Length frequency (100-nm bins), single exponential fit, parameter 240 ± 10 nm; no run lengths were measurable <400 nm. *n* = 229. **(F)** Example kymographs of rhodamine-labeled dynamic microtubules in 7 µM tubulin with 0 and 50 nM dimeric Kif18b_590_-GFP. **(G)** Measured growth speeds of dynamic extensions in the presence of 7 µM tubulin and 0, 12.5, and 50 nM dimeric Kif18b_590_-GFP. Asterisks indicate ANOVA significance: ****, P < 0.0001. **(H)** Catastrophe frequencies of dynamic microtubule extensions in the presence of 7 µM tubulin and 0, 12.5, and 50 nM dimeric Kif18b_590_-GFP. Each data point corresponds with the catastrophe frequency for an individual microtubule. Asterisks indicate Kolmogorov–Smirnov significance: ***, P < 0.001; ****, P < 0.0001. **(I)** Mean lengths of dynamic microtubule extensions in the presence of 7 µM tubulin and 0, 12.5, and 50 nM dimeric Kif18b_590_-GFP. Each data point corresponds with the mean length of the rhodamine-labeled extension from a Hilyte_647_-labeled seed over the course of a kymograph. Asterisks indicate Kolmogorov–Smirnov significance: *, P < 0.05. Error bars represent SEM.

We noted that the Kif18bΔ_C_ retained some ability to dwell at microtubule ends, suggesting that the remaining C terminus of Kif18b (591–767) contributed plus tip targeting properties to Kif18b. To exclude effects of the C terminus of Kif18b on the depolymerase activity of Kif18b, we generated a dimeric Kif18b construct, Kif18b_590_, lacking residues 591–852 (Fig. 3 A). We found that Kif18b_590_ does indeed depolymerize GMP-CPP–stabilized microtubules (Fig. 6, A and B), with a depolymerization rate of 0.046 ± 0.002 µm/min, comparable with that seen for Kif18a (0.05 µm/min; Locke et al., 2017). Although the C terminus of Kif18b inhibits its depolymerase activity in vitro, in cells the binding of this region to other partners, for example EBs, may alleviate this interference.

Single Kif18b_590_ motors walk faster and have a much shorter run length than Kif18b (Fig. 6, C–E). The mean velocity of Kif18b_590_ motors is 610 ± 35 nm/s in 0.1 mM ATP. The run length distribution can be fitted by an exponential with a decay constant *t* = 240 ± 10 nm. This shows that although CT_Kif18b_ is important for correct in vivo regulation of Kif18b, the entire C terminus contributes to enhancing the run length and enabling plus tip accumulation of Kif18b in vitro. We next observed Kif18b_590_ on dynamic microtubules. Kif18b_590_ did not reach microtubule plus ends or tip track because of its short run length (Fig. 6, F–I). However increasing concentrations of Kif18b_590_ led to a significant increase in both catastrophe frequency and growth rate. Microtubule length reduced with increasing concentration of Kif18b_590_ but remained modest.

### Kif18b promotes microtubule catastrophe

**Figure 7.**
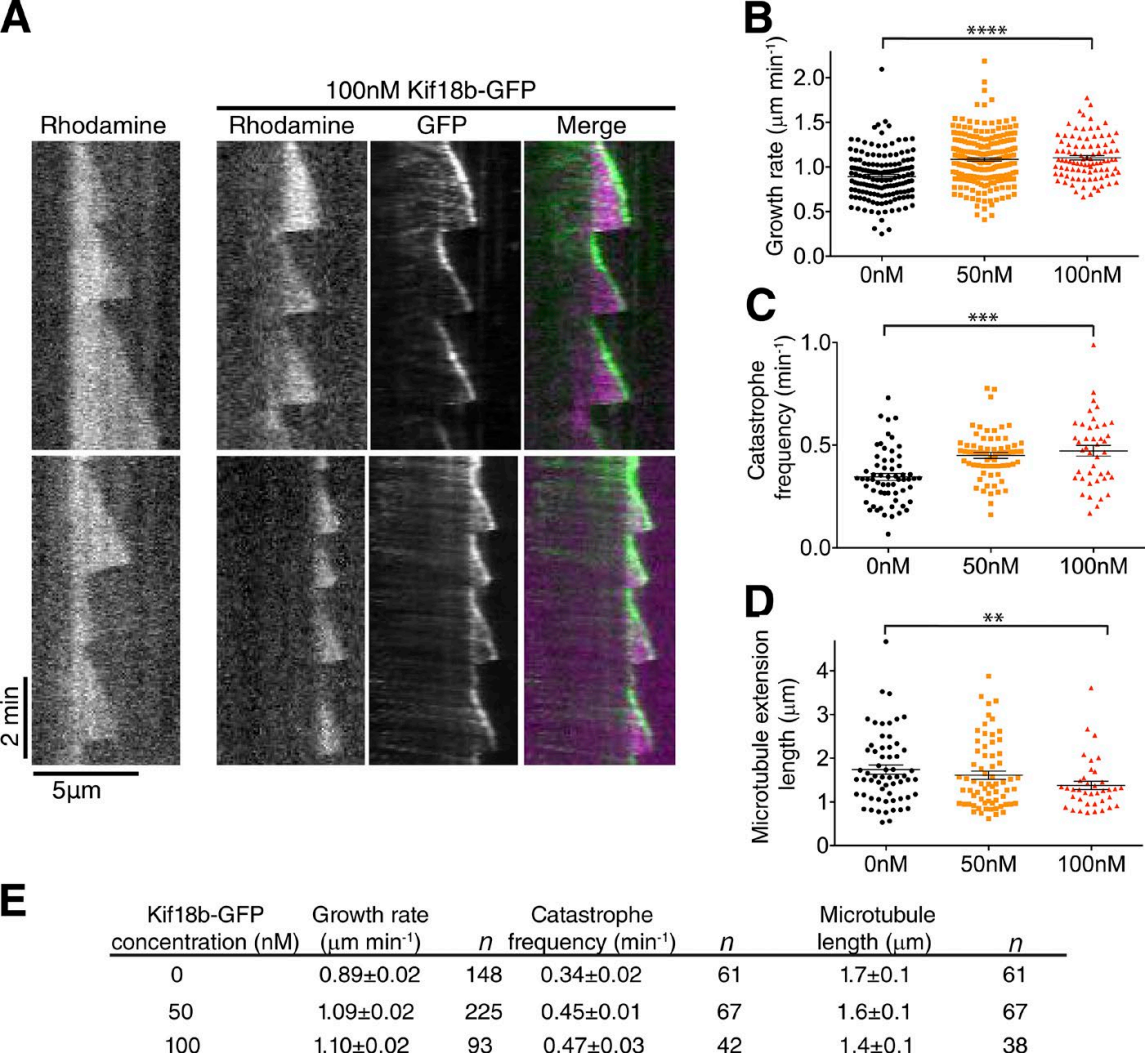
**Kif18b accumulates at the tips of growing microtubules and acts as a catastrophe factor to reduce microtubule length. (A)** Kif18b tracks the tips of growing microtubules. Example kymographs of rhodamine-labeled dynamic microtubules in 7 µM tubulin with 0 and 100 nM dimeric Kif18b-GFP. **(B)** Measured growth speeds of dynamic extensions in the presence of 7 µM tubulin and 0, 50, and 100 nM dimeric Kif18b-GFP. Asterisks indicate ANOVA significance: ****, P < 0.0001. **(C)** Catastrophe frequencies of dynamic microtubule extensions in the presence of 7 µM tubulin and 0, 50, and 100 nM dimeric Kif18b-GFP. Each data point corresponds with the catastrophe frequency for an individual microtubule. Asterisks indicate Kolmogorov–Smirnov significance: ***, P < 0.001. **(D)** Mean lengths of dynamic microtubule extensions in the presence of 7 µM tubulin and 0, 50, and 100 nM dimeric Kif18b-GFP. Each data point corresponds with the mean length of the rhodamine-labeled extension from a Hilyte_647_-labeled seed over the course of a kymograph. Asterisks indicate Kolmogorov–Smirnov significance: **, P < 0.002. Error bars represent SEM. **(E)** Table containing the microtubule dynamic parameters (mean + SEM) in the absence and presence of 50 and 100 nM Kif18b-GFP.

Although Kif18b_590_ displayed microtubule depolymerase properties, it was not able to accumulate to microtubule plus ends. Thus, to determine how full-length Kif18b regulates dynamic microtubules, we analyzed microtubule dynamic parameters and the length of dynamic microtubules in the presence and absence of Kif18b. First, we observed that Kif18b walks processively to the ends of dynamic microtubules (Fig. 7 A). Kif18b then tracks the growing ends of microtubules. The microtubule growth speed and catastrophe frequency modestly increase with increasing amounts of Kif18b (Fig. 7, B and C). Inversely, the length of dynamic microtubule extensions decreased with increasing concentrations of Kif18b (Fig. 7, D and E). Extensions in the presence of tubulin alone were a mean of 1.7 ± 0.1 µm long, whereas in the presence of 100 nM Kif18b, extensions were significantly shortened to 1.4 ± 0.1 µm (mean ± SEM). Increased Kif18b levels did not dramatically increase microtubule catastrophe; rather Kif18b was then observed on the lattice. Kif18bΔ_C_ also tracked growing microtubule ends but had a nonsignificant reduced effect on microtubule dynamics, likely because of its reduced residency time at microtubule ends (Fig. S5). Overall, these results indicate that full-length Kif18b causes an increase in microtubule dynamicity in vitro, which leads to a modest reduction in microtubule length. The C terminus of Kif18b is essential for Kif18b plus tip targeting; however, it also interferes with its depolymerase activity in vitro.

In total, our data demonstrate that Kif18b uses its catalytic and motile properties to promote remodeling of the cytoskeleton during mitosis and positioning of the metaphase spindle. Kif18b is a processive plus end–directed motor that uses its C terminus to accumulate at microtubule ends, where it promotes microtubule catastrophe. Mitotic phosphorylation of CT_Kif18b_ provides a mechanism to regulate the accumulation of Kif18b to subpopulations of microtubule ends, depending on the local kinase-phosphatase environment, and to shorten subcellular populations of microtubules to ensure spindle positioning. As its effect in vitro on microtubule length is weak, Kif18b may also use activation partners or its motile properties to transport other microtubule regulators and shorten microtubules.

## Discussion

Microtubule depolymerization is an essential process in eukaryotes to ensure microtubule remodeling and facilitate microtubule-based processes. Kinesin-13 and kinesin-8 are the major motor families responsible for stimulating microtubule depolymerization. Although the mechanisms by which kinesin-13 motors destabilize microtubule ends is well established, the mechanism by which kinesin-8 motors reduce microtubule length is more controversial and differs between members across species. Recent work published that Kif18b is a poorly processive motor that uses diffusion to reach microtubule ends, suggesting that Kif18b tracks microtubule plus ends through a bind-and-release mechanism, similarly to other plus tip tracking proteins (Akhmanova and Steinmetz, 2008; Shin et al., 2015). We believe that, in these experiments, the N-terminal GFP tag inhibits the motile properties of Kif18b, leading to its diffusive behavior (Fig. S1 F; Shin et al., 2015). Instead, our data reveal that Kif18b is a highly processive motor that walks to the plus ends of microtubules and negatively regulates dynamic microtubules by promoting catastrophe. The single-molecule behavior of Kif18b, with high processivity and dwell times at microtubule tips reported in this study, is similar to that of kinesin-8s Kif18a and yeast Kip3, although Kif18b is faster (Stumpff et al., 2011; Su et al., 2011). As with Kif18a and Kip3, the run length and microtubule tip dwell time of Kif18b is enhanced by the presence of a second microtubule-binding region. On stable microtubules, the rate of depolymerization of Kif18b_590_ is similar to that of the Kif18a motor domain as would be expected given the similarity in sequence within the two motor domains. However, the Kif18b C terminus interferes with this depolymerization activity. Interestingly the tail domain of Kip3 has also been shown to counteract the depolymerase activity of Kip3, by stabilizing shrinking microtubules (Su et al., 2011). Future work is needed to examine whether the C terminus of Kif18b interferes with the depolymerase activity of the Kif18b motor domain when EB proteins and other Kif18b-regulatory proteins are present.

We also show that unlike Kif18a, Kif18b does not appear to increase pausing and microtubule stabilization (Stumpff et al., 2011). Although Kif18a appears to dampen microtubule dynamics, we demonstrate that Kif18b achieves a reduction in microtubule length through an increase in microtubule dynamics, principally the catastrophe frequency (Stumpff et al., 2011). The C termini of Kif18b and Kif18a are highly divergent and explain the distinct targeting properties. Additionally, the loop L2 is significantly different in Kif18a, Kif18b, and Kip3. In kinesin-13, this loop contains the KVD motif, which is essential for microtubule depolymerization. Therefore, this region could also be responsible for biochemical differences between kinesin-8s.

Kif18b uses a C-terminal nonmotor microtubule-binding domain to recognize and accumulate at microtubule ends in vitro*,* similar to multiple other kinesins (Welburn, 2013). These nucleotide-insensitive microtubule-binding regions play important roles in specifying the unique functions and targeting of kinesins. Kif18b_590_ lost its ability to track growing microtubules and had a severely shortened run length. Kif18bΔ_C_ lacking its regulatory CT_Kif18b_ retained some ability to dwell at microtubule plus ends in vitro. However, in the crowded cellular context where other proteins compete for microtubule plus-end binding, CT_Kif18b_ seems critical for plus tip accumulation. Given its long run length and its speed in vitro, Kif18b may walk along microtubules in vivo until it reaches microtubule ends. Single-molecule imaging of Kif18b in vivo measured that Kif18b moves processively on microtubules at a speed of 635 nm/s, which would allow it to reach the end of a polymerizing microtubule (Tanenbaum et al., 2014). We show in this study that in vitro, Kif18b can track growing ends autonomously. In vivo, Kif18b appears to use a dual plus end–targeting mechanism, through microtubule end tethering and EB binding, to robustly localize to the growing microtubule tip (Fig. S5 F). Endogenous Kif18b is enriched specifically at the ends of astral microtubules and is excluded from microtubules in the vicinity of chromosomes and centrosomes (Stout et al., 2011; Tanenbaum et al., 2011). Aurora kinases and Plk1 localize specifically to chromosomes and centrosomes during mitosis, thereby creating a spatial phosphorylation gradient for substrates (Liu et al., 2009; Ye et al., 2015). We propose that phosphorylation of the CT_Kif18b_ by spatially restricted mitotic kinases could exclude or at least reduce Kif18b from dwelling at the tips of microtubules in these regions. As Kif18b walks on microtubules away from the kinase activity gradient, it becomes dephosphorylated by cytoplasmic phosphatases. This dephosphorylation allows Kif18b to dwell at microtubule tips longer, leading to subcellular accumulation of Kif18b at the plus tips of the longer astral microtubules. In this manner, microtubule plus end–localized Kif18b will be able to selectively destabilize these microtubules, helping to ensure correct spindle positioning. In vitro, CT_Kif18b_ is not an Aurora kinase substrate (unpublished data). Several kinases and phosphatases control spindle positioning by regulating how the LGN–NuMA complex recruits the cortical force generator dynein (Kiyomitsu and Cheeseman, 2012; Afshar et al., 2016; Kotak et al., 2016). Future work should determine which mitotic kinases and phosphatases regulate Kif18b spatially and temporally to control microtubule length and spindle positioning close to the cortex. Changes in astral microtubule length alter the contacts between the spindle and the cortical force generators and the length-dependent forces applied to the spindle, which causes mispositioning (Howard and Garzon-Coral, 2017). We propose that Kif18b controls spindle positioning by shortening microtubules, thereby preventing excessive contacts between astral microtubules and the cell cortex, rather than directly affecting the cortical LGN–NuMA complex.

Our study sheds light on at least one mechanism by which Kif18b destabilizes microtubules. Kif18b regulates microtubule plus end dynamics negatively by promoting catastrophe, resulting in a net reduction in the length of microtubules (Fig. 7). Because Kif18b also modestly increases microtubule growth speed, microtubules are not completely depolymerized, unlike in the presence of MCAK. Rather, microtubules have a tighter length distribution, which is important in mitosis. Kif18b is nuclear in interphase. In mitosis, Kif18b localizes preferentially to a subset of microtubules extending toward the cell cortex (Tanenbaum et al., 2011; Walczak et al., 2016). Therefore, Kif18b access to microtubules at mitotic onset and its spatial regulation contribute to the increase in microtubule dynamics and remodeling of the cytoskeleton in mitosis. In agreement with previous research, our data suggest that MCAK and Kif18b may work in the same pathway to regulate microtubule length (Tanenbaum et al., 2011). However, this may differ with cell types and organisms (Rankin and Wordeman, 2010; Corrigan et al., 2013; Garzon-Coral et al., 2016). Future work should determine the mechanism by which Kif18b and MCAK cooperate using their distinct mechanisms to regulate microtubules during mitosis.

## Materials and methods

### Cloning

To assay the localization in cell culture of Kif18b subdomains, various constructs were generated from Kif18b transcript variant 1 (NM_001252506.1) and cloned into pBABE-puro containing an N- or C-terminal LAP tag using restriction enzymes (Cheeseman and Desai, 2005). For constructs expressing a C-terminal LAP-tag at the C terminus of Kif18b, an N-terminal Kozak consensus sequence (5′-ACCGTCGCCAC-3′) was included upstream of Kif18b for enhanced translation initiation. The nonphosphorylatable (SA) and phosphomimetic (SD) mutants were synthesized as gBlock fragments (Integrated DNA Technologies). The CT_Kif18b_ and the corresponding mutants CT_Kif18b_SD and CT_Kif18b_SA were cloned into pET3aTr and included a 3C protease cleavage site followed by a 6xHis tag. Full-length Kif18b_SD_ and Kif18b_SA_-GFP constructs were generated in a two-step PCR reaction. In the first step, the Kif18b_SD_ and Kif18b_SA_ gBlock fragments and Kif18bΔ_C_ were amplified with the following sets of overlapping primers: 5′-ccc*GTCGA*CACCGTCGCCACCATGGCAGTGGAGGACAGCACGC-3′, 5′-TTGGCTTGGGGCCCTTCATGGTGAACAGGGGCACAGGTGCCCTGG-3′ and 5′-CTGTGCCCCTGTTCACCATGAAGGGCCCCAAGCCAACATCTT-3′, 5′-ccc*GAATTC*GACACCTTGGTGACGCCGTTC-3′, respectively. In the second step, the fragments were joined by PCR using overlapping overhangs created by the primers in step 1 using 5′-ccc*GTCGAC*ACCGTCGCCACCATGGCAGTGGAGGACAGCACGC-3′, 5′-ccc*GAATTC*GACACCTTGGTGACGCCGTTC-3′. In all sequences, the primer overhang and the enzyme restriction sites are highlighted in lowercase and uppercase italics, respectively. The final PCR product was then inserted into a pBABE-puro–derived plasmid using SalI and EcoRI. Previously proposed EB-binding motifs were mutated using site-directed mutagenesis (QuikChange). Residues mutated are underlined: _653_SFLP_656_, _773_SSLP_776_, and _799_ARLP_802_, although we do not consider this last sequence an SXIP motif as A at this position is nonbinding to EB (Buey et al., 2012). For recombinant expression in insect cells, full-length Kif18-GFP-6×His was cloned into pFL. Details of constructs and cloning are listed in Table S1.

### Protein expression, purification, and assays

Protein purification and microtubule cosedimentation assays were performed as previously described (Talapatra et al., 2015). His-tagged proteins were purified using Ni-NTA–agarose beads (GE Healthcare) according to the manufacturer’s guidelines. Proteins were then purified using gel filtration chromatography preequilibrated in gel filtration buffer (for full-length Kif18b: 25 mM Hepes, pH 7.5, 150 mM NaCl, 300 mM KCl, 5 mM β-mercaptoethanol, 1 mM MgCl_2_, 1 mM Na-EGTA, and 1 mM ATP; for Kif18b microtubule-binding domain: 50 mM Hepes, pH 7.5, 150 mM NaCl, and 5 mM β-mercaptoethanol). Analytical gel filtration chromatography was performed using either a Superdex 75 or a Superdex 200 10/300 GL column (GE Healthcare). For the Kif18b microtubule-binding domain, cleavage of the 6xHis tag was performed using the his-3C protease overnight at 4°C. For detection of Kif18b by Western blotting, an antibody against Kif18b_500–593_ was raised in rabbit (Covance) and affinity purified.

### SEC-MALS

SEC-MALS was performed using a Superdex 200 10/300 GL column on an ÄKTA fast protein liquid chromatography system. MALS measurements were performed using a MiniDAWN in-line detector (Wyatt Technology). Kif18b-GFP-His was at 0.3 mg/ml in gel filtration buffer described above. Kif18b_590–593_ was at 2 mg/ml. Protein concentration was monitored using a UV monitor at 280 nm, and a refractive index detector was set at 690 nm (Optilab DSP; Wyatt Technology). Data were analyzed as previously described (Talapatra et al., 2015).

### Cell culture and transfection

HeLa cells were used and maintained in DMEM (Lonza) supplemented at 37°C in a humidified atmosphere with 5% CO_2_. Cells were monthly checked for mycoplasma contamination (MycoAlert detection kit; Lonza). For live imaging, cells were plated on 35-mm glass-bottom microwell dishes (MatTek). Transient transfections were conducted using Effectene reagent (QIAGEN) according to the manufacturer’s guidelines. RNAi experiments were conducted using RNAi MAX transfection reagent (Invitrogen) according to the manufacturer’s protocol. Cells were visualized after 24–48 h. Previously published and validated siRNAs were used to deplete Kif18b (Kif18b ON-TARGETplus SMARTpool siRNA; GE Healthcare; Tanenbaum et al., 2011). Control RNAi was a siGENOME nontargeting siRNA pool 1 (GE Healthcare).

### CRISPR-Cas9 KO

The Kif18b single guide RNA was designed using http://crispr.mit.edu. Phosphorylated oligonucleotides 5′-CACCCACGCTGCAAGTAGTGGTAC-3′ and 5′-AAACGTACCACTACTTGCAGCGTG-3′ were annealed and ligated into pX330. We transfected mCherry–*Streptococcus pyogenes* Cas9 (gift from P. Hohenstein, University of Edinburgh, Edinburgh, Scotland, UK) and the plasmid pX330 (gift from F. Zhang, Broad Institute, Cambridge, MA) containing a single guide RNA targeting the first exon of the Kif18b gene in HeLa cells. Double-stranded DNA breaks are generated in the targeted exon such that repairs of these cuts can generate indels that disrupt the open reading frame and abolish protein synthesis (Cong et al., 2013). We then selected the monoclonal cell lines that lacked Kif18b and verified that the depletion was specific to Kif18b. After 48 h, single cells were sorted into 96-well plates. Clonal cell lines were screened by Western blot for Kif18b depletion.

### Microscopy

For live-cell imaging, HeLa cells were transferred to Leibowitz L15 medium (Thermo Fisher Scientific) supplemented with 10% FBS and penicillin/streptomycin (Gibco) and imaged at 37°C using a DeltaVision core microscope (Applied Precision Ltd.) equipped with a CoolSnap HQ2 charge-coupled device camera. For live-cell imaging, 4–10 z sections were acquired at 0.5–1-µm steps using a 60× or 100× objective lens. For live imaging of spindle positioning, the cells were imaged every 3 min. To visualize microtubules and the cell cortex, 20–50 nM SiR dye (SpiroChrome) and 1:2,000 to 1:40,000 dilution of Cell Mask orange (Thermo Fisher Scientific) were used for 3 h and 5 min, respectively. For fixed imaging, 10–20 z sections were acquired at 0.2–0.5 µm. For immunofluorescence, cells were then washed in PBS and fixed by one of two methods: either in methanol for 10 min at −20°C and permeabilized with cooled acetone for 1 min at –20°C, or in 3.8% formaldehyde in PHEM buffer (60 nM Pipes, 25 mM Hepes, 10 mM EGTA, and 2 mM MgSO_4_, pH 7.0) for 20 min. Immunofluorescence in human cells was conducted using antibodies against mouse anti–β-tubulin (1:1,000; Sigma-Aldrich), mouse EB1 (1:400; BD), and rabbit Ndc80 antibodies (1:1,000; gift from I. Cheeseman, Massachusetts Institute of Technology, Cambridge, MA). For experiments with small-molecule inhibitors, the proteasome inhibitors MG132, ZM447439, and MLN8237 and the Eg5 inhibitor STLC were used at final concentrations of 10 µM, 2 µM, 300 nM, and 5 µM, respectively. For examining levels of Kif18b at plus tips, we imaged transfected Kif18b-GFP and EB1. Comet intensities stained for EB1 were measured using a linescan in MetaMorph. A linescan parallel and close to the comet was used as background fluorescence and subtracted from the comet intensity. The linescans were then overlaid to measure the intensities for the GFP constructs at the same location. Comet mean intensity for EB1 and GFP constructs was obtained by aligning EB1 comet profiles with respect to the maximum EB1 peak intensity. Individual linescans for Kif18b-GFP constructs were then aligned with the translation corresponding with its matched EB1 linescan, and mean intensities were calculated for Kif18b-GFP constructs. In Fig. 3, GFP intensities were binned into four classes (low, medium, high, and overexpression). Analysis was performed on low- and medium-expressing cells. Only cells expressing low levels of Kif18b were analyzed to avoid artifacts resulting from overexpression. For microtubule length measurement and GFP signal visualization, 20 and three z sections were acquired at 0.2–0.3-µm and 1-µm step sizes, respectively.

### TIRF microscopy

For experiments using stabilized microtubule seeds, silanized coverslips were prepared as by Bechstedt et al. (2011) except that in place of treatment with Piranha solution, coverslips were incubated overnight in 12% hydrochloric acid at 50°C. For experiments using dynamic microtubules, coverslips were prepared with a with a PEG-biotin surface (Bieling et al., 2010). Flow chambers were formed using double-sided tape, a treated coverslip, and a microscopy slide. Flow chambers were 7–8 µl in volume.

All experiments were performed in BRB80 buffer (80 mM K ⋅ Pipes, 1 mM MgCl_2_, and 1 mM EGTA, pH 6.8). For single-molecule assays, anti–β-tubulin antibodies (Sigma-Aldrich) at 1:10 dilution were first introduced to the chamber. The surface was then blocked with 1% pluronic F-127 (Sigma-Aldrich), and 7% rhodamine-labeled microtubules (Cytoskeleton) stabilized with GMP-CPP (Jena Bioscience) were bound to the glass surface via the antibodies. The surface was then further blocked with 1 mg/ml casein (Sigma-Aldrich assay buffer consisting of BRB80 with 1 mM ATP, 0.5 mg/ml casein, and an oxygen-scavenging system [0.2 mg/ml glucose oxidase, 0.035 mg/ml catalase, 4.5 mg/ml glucose, and 140 mM β-mercaptoethanol]). 1–10 nM dimeric Kif18b-GFP-His, Kif18bΔ_C_-GFP-His in assay buffer was introduced to the flow chamber, and the chamber was sealed with nail varnish and imaged immediately at 37°C. For Kif18b_590_-GFP-His single-molecule experiments, an ATP concentration of 0.1 mM and a temperature of 30°C were used.

For dynamic microtubule experiments, the surface was first blocked with 5% pluronic F-127 before introduction of 50 µg/ml NeutrAvidin (Invitrogen). The chamber was washed with 1 mg/ml casein and 1% Tween-20 (Scientific Laboratory Supplies) before introduction of GMP-CPP–stabilized microtubule seeds with 7% Hilyte_647_ label (Cytoskeleton) and 7% biotin label (Cytoskeleton). Microtubule seeds were allowed to bind to the surface before further blocking with 1 mg/ml casein and 1% Tween-20. Final assay buffer consisted of BRB80 with 1 mM ATP, 1 mM GTP, 0.5 mg/ml casein, 0.5% Tween-20, 7 µM tubulin (6% rhodamine-label), and up to 250 nM motor mix (or an equivalent volume of gel filtration buffer) and an oxygen scavenging system. The assay buffer was introduced to the flow chamber, and the chamber was sealed with nail varnish and imaged at 30°C.

Imaging was performed on a Zeiss Axio Observer Z1 TIRF microscope using a 100× NA1.46 objective and a Photometrics Evolve Delta electron-multiplying charge-coupled device camera controlled by Zeiss Zen Blue software. Single-molecule imaging was performed for up to 5 min at 2 frames per second (fps) for Kif18b-GFP-His and Kif18bΔ_C_-GFP-His, and for up to 1 min at 10 fps for Kif18b_590_-GFP-His. For calculation of tip dwell times, a frame rate of 1 fps was used. Depolymerization assays were performed over 15 min at 4 fpm. Microtubule dynamics were imaged at 0.25 fps for up to 15 min. Kymographs were produced using ImageJ (National Institutes of Health), and run lengths and velocities of motors and microtubule growth rates were measured from these kymographs. Tip dwell times were calculated from two-color kymographs where a motor could be seen to reach an unoccupied microtubule tip. Catastrophe frequencies were calculated from the length of and number of catastrophes seen in individual kymographs. Microtubule extension lengths are means over individual kymographs. Normality was checked using Anderson–Darling test, and P < 0.05 was used to reject the null hypothesis. Kolmogorov–Smirnov test was used to compare data that were not normally distributed (Fig. 6, H and I; Fig. 7, C and D; and Fig. S5, C and D). Images were stored and visualized using an OMERO.insight client (OME; Allan et al., 2012).

### Statistics and reproducibility

Statistical analyses were performed using Prism 6.0 (GraphPad Software) or R software. No statistical method was used to predetermine sample size. No samples were excluded from the analyses. The investigators were not blinded to allocation during experiments and outcome assessment. All experiments were performed and quantified from at least three independent experiments (unless specified otherwise), and representative data are shown.

### Online supplemental material

Fig. S1 shows the effect of kinesin-8 and kinesin-13 motor families on microtubule length during mitosis, the effect of Kif18b RNAi depletion on spindle positioning, and how a GFP N-terminal tag interferes with Kif18b localization. Fig. S2 shows the purification of Kif18b C-terminal constructs. Fig. S3 shows that Kif18b_SXIPmut2_ still targets to microtubule plus ends and that Kif18b-GFP rescues spindle architecture defects. Fig. S4 shows the biochemical characterization of Kif18b. Fig. S5 shows how Kif18bΔ_C_ accumulates at the tips of growing microtubules and its effect on microtubule dynamics. Video 1 shows a cell in metaphase displaying rocking and displacement of the spindle over time. Video 2 uses single-molecule imaging to show Kif18b-GFP walking toward the plus ends of microtubules. Table S1 lists the constructs and cloning details used in this study.

## Supplementary Material

Supplemental Materials (PDF)

Table S1 (Excel)

Video 1

Video 2
