# Supplementary material for: Microtubule end tethering of a processive kinesin-8 motor Kif18b is required for spindle positioning
Source: J Cell Biol. 2018 Jul 2;217(7):2403–16. doi: 10.1083/jcb.201705209 (PMC6028548; doi:10.1083/jcb.201705209)
Supplement: Supplemental Materials (PDF) [file JCB_201705209_sm.pdf]

JCB

Supplemental material

McHugh et al., https://doi.org/10.1083/jcb.201705209

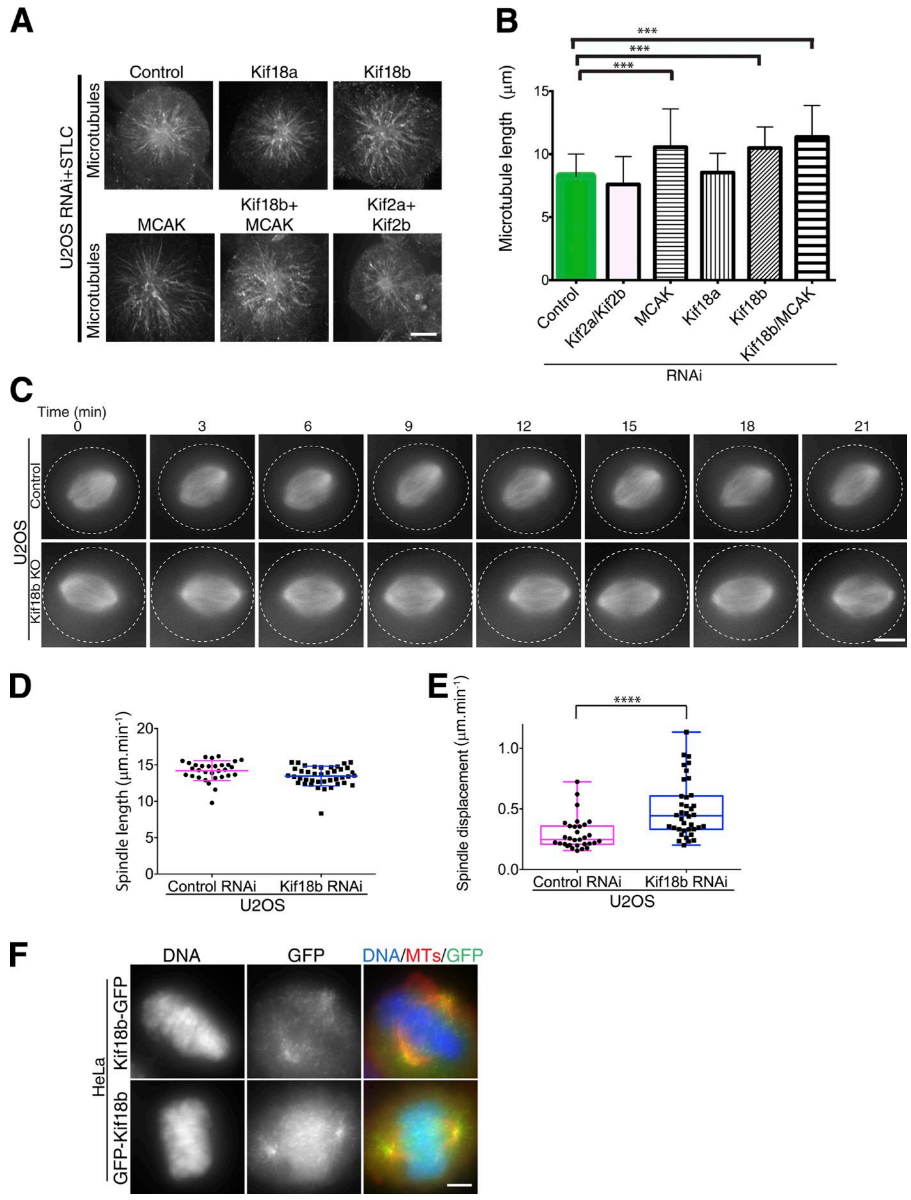

Figure S1. **Effect of kinesin-8 and kinesin-13 motor families on microtubule length during mitosis. (A)** Immunofluorescence images acquired using a β-tubulin antibody in U2OS cells depleted with control, kinesin-8, or kinesin-13 siRNA and treated with STLC. **(B)** Bar graph showing the mean microtubule (MT) length for microtubules radiating from the center of the aster (mean ± SD) for depletions in A. Asterisks indicate ordinary one-way ANOVA significance: ***, P < 0.001. Experiment quantified once. **(C)** Representative time-lapse imaging of U2OS cells expressing mCherry-tubulin treated with control or kif18b RNAi in the presence of the protease inhibitor MG132. **(D)** Graph representing the mean spindle length and the corresponding SD for control- and kif18b RNAi–treated U2OS cells (*n* = 31 and 39). **(E)** Box-and-whisker plot showing quantification of the displacement of the spindle from the center of the cell during metaphase. Each point represents the displacement of one spindle over at least 30 min. Line in the middle box is plotted as the median, edges of the box represent 25th and 75th percentiles, and whiskers represent the minimum and maximum displacement of cells. Asterisks indicate unpaired *t* test significance: ****, P < 0.0001. **(F)** Representative images of GFP-Kif18b– and Kif18b-GFP–transfected HeLa cells costained with DNA and a β-tubulin antibody. Bars, 5 µm.

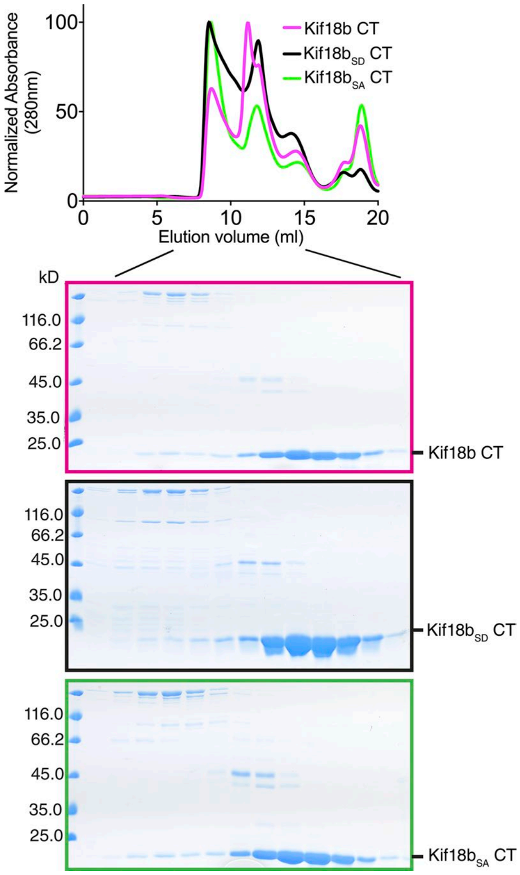

Figure S2. **Purification of Kif18b C-terminal constructs.** SEC elution profiles of the purified Kif18b$_{767–852}$ (pink), Kif18b$_{767–852}$SD (black), and Kif18b$_{767–852}$SA (green) with corresponding Coomassie-stained gels.

JCB

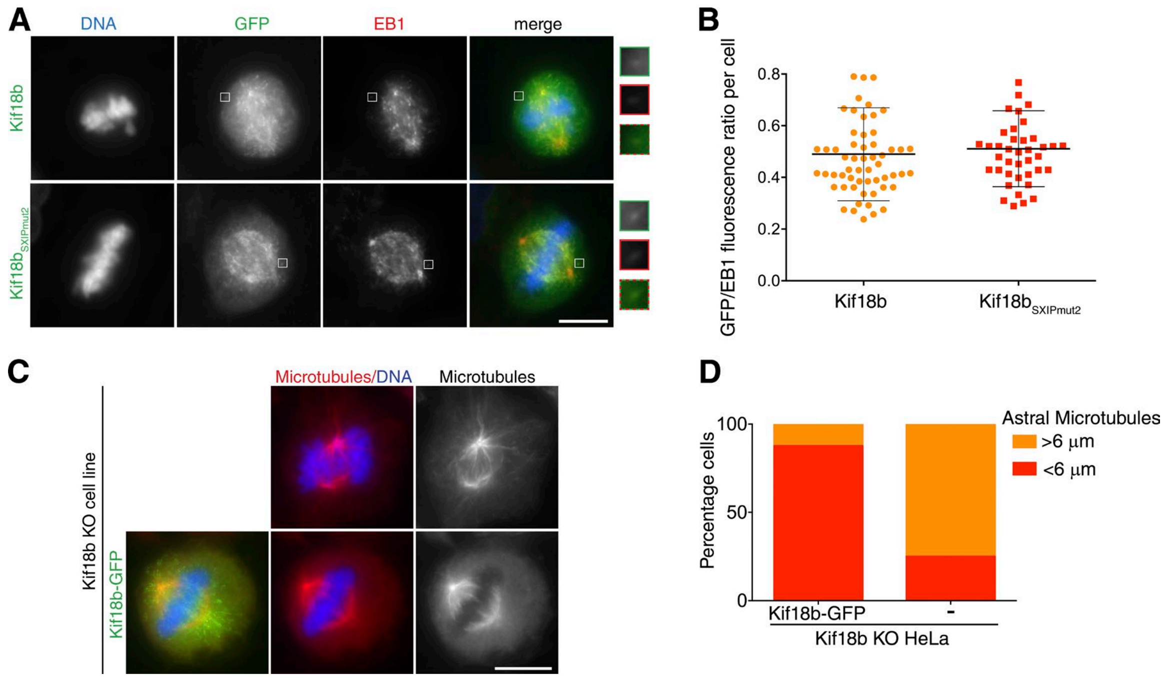

Figure S3. **Regulation and function of Kif18b on microtubules. (A)** Representative immunofluorescence images of Kif18b-KO HeLa cells transfected with Kif18b$_{SXIPmut2}$-GFP construct and stained for EB1 and DNA. **(B)** Quantification of GFP/EB1 signal intensity ratio at microtubule plus end for Kif18b-GFP and Kif18b$_{SXIPmut2}$-GFP (mean ± SD). Each data point represents one cell for which usually four comet intensities were measured and averaged. **(C)** Representative images of Kif18b KO cells transfected with Kif18b-GFP and costained with a β-tubulin antibody and DNA. **(D)** Quantification of astral microtubule length in the Kif18b KO cell line in the absence and presence of Kif18b-GFP (n = 52 and 46, respectively). Bars, 10 µm.

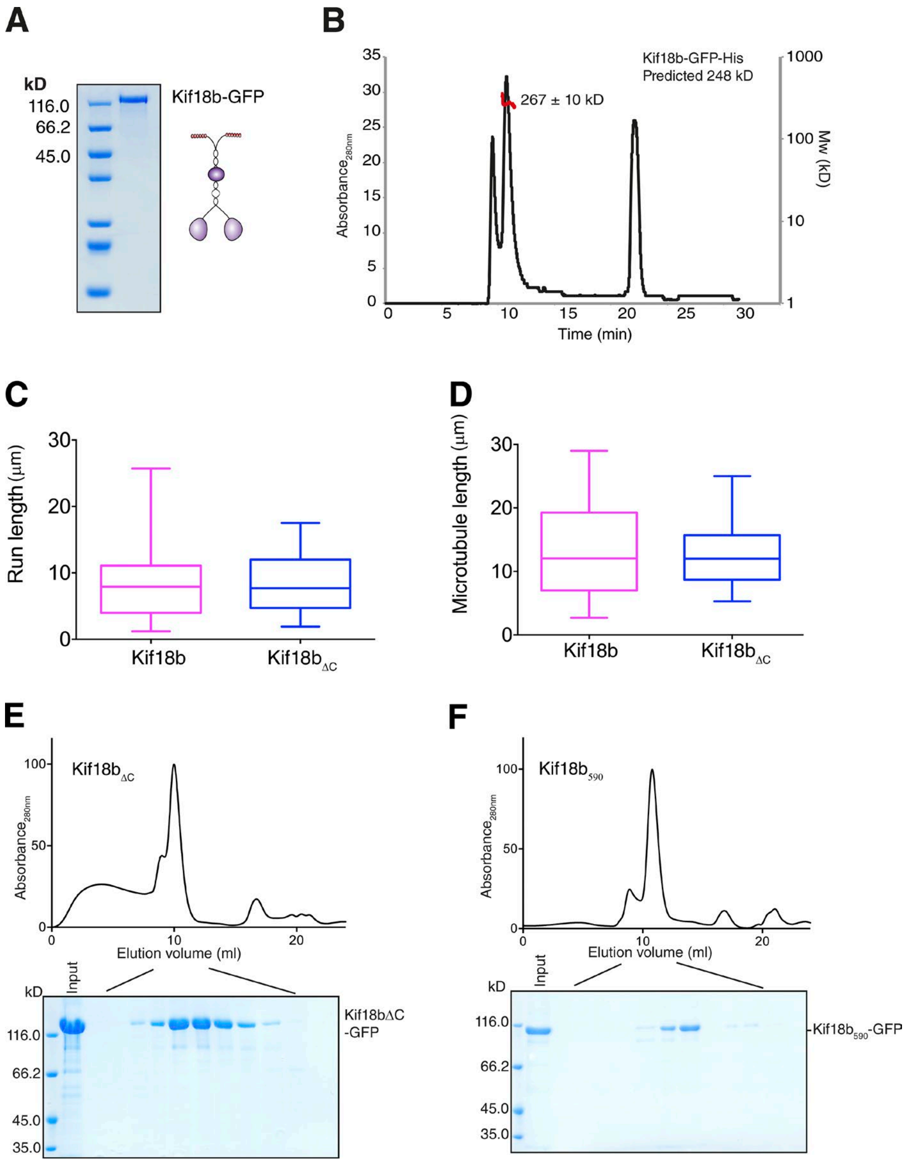

Figure S4. **Biochemical characterization of Kif18b. (A)** Coomassie-stained gel of insect cell–expressed and purified Kif18-GFP. **(B)** Kif18b-GFP is dimeric in solution. Elution profile for Kif18b-GFP-His (black line, left y axis) from SEC-MALS. Outcome of the MALS analysis for the peaks is presented in red (molecular weight [Mw], right y axis). Molecular weight ± uncertainty is indicated. Secondary peak at time ~20 min corresponds with excess GFP. **(C)** Coomassie-stained gel showing SEC elution of Kif18bΔ$_C$-GFP-His (top) with corresponding SEC elution profile of the purified motor (bottom). **(D and E)** The run length of Kif18b (*n* = 44; median 7.9 µm) and Kif18bΔ$_C$ (*n* = 33; median 7.7 µm) is limited by microtubule length, with very few motors detaching from the microtubule before reaching the microtubule end. No significant difference can be seen in the run length between the two motors (B). The motor run lengths for the two motors were measured over similar microtubule populations. **(F)** Coomassie-stained gels showing SEC elution of Kif18b$_{590}$-GFP-His (top) with corresponding SEC elution profile of the purified motors (bottom).

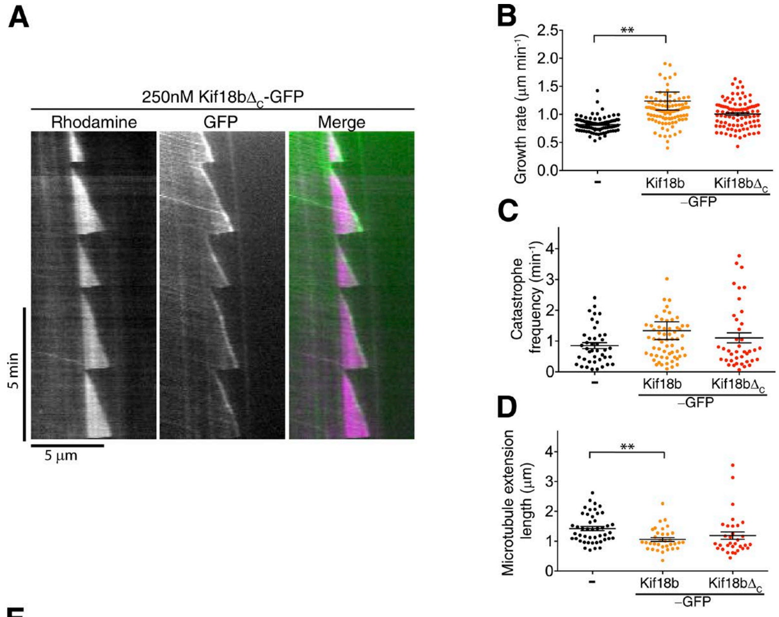

E

	Growth rate (µm min-1)	*n*	Catastrophe frequency (min-1)	*n*	Microtubule length (µm)	*n*
Microtubules alone	0.82±0.01	88	0.85±0.06	41	1.42±0.07	46
Kif18b-GFP(250nM)	1.09±0.02	95	1.10±0.02	40	1.06±0.06	32
Kif18bΔc-GFP(250nM)	1.00±0.02	106	1.06±0.08	60	1.18±0.12	34

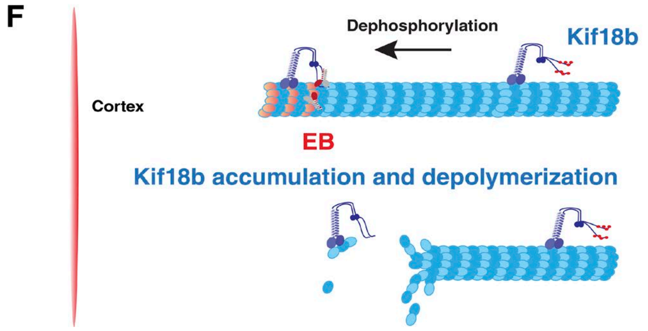

Figure S5. **Kif18bΔ$_C$ accumulates at the tips of growing microtubules. (A)** Kif18bΔ$_C$ tracks the tips of growing microtubules. Kymograph of rhodamine-labeled dynamic microtubules in 7 µM tubulin with 0 and 100 nM dimeric Kif18b-GFP. **(B)** Measured growth rates of dynamic extensions in the presence of 7 µM tubulin and 250 nM dimeric Kif18b-GFP and Kif18bΔ$_C$-GFP. Asterisks indicate ANOVA significance: **, P < 0.01. **(C)** Catastrophe frequencies of dynamic microtubule extensions in the presence of 7 µM tubulin and 0, 50, and 100 nM dimeric Kif18b-GFP. Each data point corresponds with the catastrophe frequency for an individual microtubule. **(D)** Mean lengths of dynamic microtubule extensions in the presence of 7 µM tubulin and 0, 50, and 100 nM dimeric Kif18b-GFP. Each data point corresponds with the mean length of the rhodamine-labeled extension from a Hilyte$_{647}$-labeled seed over the course of a kymograph. Asterisks indicate Kolmogorov–Smirnov significance: **, P < 0.002. Error bars represent SEM. **(E)** Table containing the microtubule dynamic parameters (mean + SEM) in the absence and presence of 250 nM 18b-GFP and Kif18bΔ$_C$-GFP. **(F)** Schematic model of Kif18b walking toward the plus end of a microtubule. Kif18b is phosphorylated close to centrosomes and chromosomes. As Kif18b reaches the cortex, the C terminus is dephosphorylated by cytoplasmic or cortical phosphatases, which allows Kif18b to accumulate at the ends of astral microtubules to destabilize them. Kif18b and MCAK could cooperate at microtubule plus ends to destabilize microtubules.

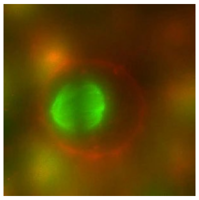

Video 1. **A cell in metaphase displaying rocking and displacement of the spindle over time.** The frame rate is one frame per 3 min.

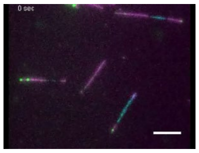

Video 2. **Single-molecule imaging of Kif18b-GFP walking toward the plus ends of microtubules.** Microtubule seeds are labeled in blue (tubulin $HiLyte_{647}$), and seeds were extended using rhodamine-tubulin to determine the polarity of microtubules. The frame rate is two frames per second. The playback rate is five frames per second or 2.5× real time.

Table S1 is a separate Excel file showing a list of constructs and cloning details used in this study.
